# Monkeypox re-emergence regulation in Ukraine through a strategic response plan: An update

**DOI:** 10.1097/JS9.0000000000000023

**Published:** 2023-01-27

**Authors:** Sumira Malik

**Affiliations:** Amity Institute of Biotechnology, Amity University Jharkhand, Ranchi, Jharkhand, India

*Dear Editor,*

The COVID-19 pandemic has imposed a global threat to all the developed and developed countries targeting social, economic, and political platforms influencing the global interconnections in 2020. In the era of ongoing long-term COVID-19 pandemic post-effects worldwide, spontaneous, and unexpected monkeypox outbreaks have attained global consideration. At the global platform, apart from its native origin central and western Africa reported monkeypox as sporadic, exceptionally huge, and massive 3340 confirmatory numbers of monkeypox epidemic were reported on 22 June 2022, at the global level. Monkeypox virus (MPXV) is a member of the *Orthopox* genus, spreads through zoonosis and in habitats rodents, mammals, and primates as an animal reservoir making it more susceptible to transmission and infective^1^. It is transmitted from animal to human through lesions, bites, direct blood contact, and treatment of wild infected animals and occasionally. Another mode is human-to-human direct contact during close contact between people through fluid exchange (vaginal or salivary) during intercourse or oral acts through touching body parts infected with the MPXV^2^,^3^. Epidemiologically, monkeypox lasts for 2–4 weeks, with the reported endemic case fluctuating fatality rate from 0 to 11% with pooled study data of 8.7% case fluctuating fatality rate^4^. The symptoms of monkeypox disease include headaches, genital, and perianal ulcerative lesions, anogenital rashes, fever, swollen lymph nodes, and pain during swallowing^5^.

In 2022, a total of 3413 confirmed cases with one death reported from 1 January 2022, to 22 June 2022, with 86% of cases from the European region from 50 countries in five WHO regions. Among these 3413 total cases from 3 May 2022, to 22 June 2022, 3340 cases were identified from nonendemic MPXV of 42 Member States in four WHO regions^6^.

Currently, as an emergency is declared in Ukraine, after the 6-month war, the country is badly affected economically and socially. The COVID-19 pandemic is under surveillance in Ukraine, however, another epidemic monkeypox is reported in Ukraine on 15 September 2022. Ukraine has declared no historical case of MPXV case in its history as per Centers for Disease Control and Prevention (CDC). According to the announcement of the health ministry, the Ukrainian health system is taking lessons in providing life-saving care with the support of WHO and partnered for combating these challenges ahead. Ukraine has reported a total of five confirmed cases of monkeypox as of 15 October 2022 (https://www.cdc.gov/poxvirus/monkeypox/response/2022/world-map.html). In Ukraine, the first incidence of monkeypox was validated in a laboratory on 15 September 2022 (https://moz.gov.ua/article/news/v-ukraini-zareestrovanij-vipadok-vispi-mavp). The current situation in Ukraine reported no introduction of mass vaccinations as per reduced registered cases of monkeypox as per WHO. However, in July as per global humanitarian help for the diagnosis of MPXV, 5000 PCR kits were delivered to Ukraine and aided in diagnosis regionwide (https://www.manilatimes.net/2022/09/17/news/world/ukraine-records-first-monkeypox-case/1858715). The infection was diagnosed owing to PCR research undertaken by the Regional Centre for Disease Control and Prevention of Ukraine’s Ministry of Health. This case is now being investigated epidemiologically. The patient claimed that he had not gone outside of the country and had no encounters with monkeypox sufferers. Nonetheless, the advent of infectious symptoms and the initial findings of the epidemiological records indicate that the individual was affected in one of the nation’s major cities. The patient was found to have an elevated body temperature and rashes on his body as symptoms of the infection. Doctors in Ukraine follow recognized WHO standards and international methods to diagnose and treat monkeypox. Given that medications are inaccessible to the general population, it is essentially the subject of symptomatic therapy. The Ministry of Health issued directives to all provincial Centers for Disease Control and Prevention in May on monitoring, case investigations, and contact tracing. As winter sets, WHO is ramping up its coordination with the Ministry of Health to assure that the healthcare staff is equipped with the essential skills to address rising demands (https://moz.gov.ua/article/news/v-ukraini-zareestrovanij-vipadok-vispi-mavp).

The Russo-Ukrainian conflict has resulted in several sociopolitical, financial, infrastructural, and healthcare implications. Furthermore, the consequences of this conflict, particularly in terms of medical care including inside and even outside Ukraine, will persist long after the actual war has finished. It is critical to acknowledge that the Ukrainian medical sector is already experiencing considerable stress because of the increasing cases of casualties and harmful ramifications for the contemporary economy. These vulnerabilities are likely to exacerbate the spread of certain other infections including measles, typhoid, malaria, polio, HIV, and further stimulate the outbreak of the monkeypox virus. Overcrowded refugees in confinement camps have been implicated in the fast development of such illnesses. Moreover, interruptions in vaccination campaigns in conflict zones might serve a significant part in the emergence of other contagious^7^. Unquestionably Ukrainian citizens’ health and livelihood are now in extreme danger as the frequency and severity of this disease may keep increasing worldwide in the absence of coordinated crisis response and as misinformation prevails. To strengthen the Ukrainian population’s resistance to epidemics like monkeypox and to mitigate the detrimental consequences of the war, immediate global actions and public health support are necessary. Despite the lack of specialized medications or vaccination for monkeypox infection, some nations have licensed smallpox antiviral medications and vaccines in anticipation of the monkeypox breakout. Monkeypox outbreaks across multiple countries must be quickly controlled to prevent the virus from establishing effective person-to-person propagation^8^. Recently, towards the termination of Ukraine conflict a new strategic revised plan (SRP) termed as National Health Strategic Plan ensuring stronger health system has been launched from June 2022 to December 2022 from WHO. The Ukrainian SRP emphasis on healthcare strategies of their citizens. Recently, a well-designed SRP focuses on partnership of WHO to access overall Ukrainian populations, in which the monkeypox virus affected populations could utilize health services provided by the Ukrainian government. As per Ukrainian SRP regulations focuses on open border policies for the admittance of Ukrainian refugees and availability of the healthcare services to infected individuals. The financial aid, human resources in enormous capacity are readily available and well sustained for the Ukrainian refugees^9^.

## Ethical approval

Not applicable.

## Sources of funding

None.

## Authors’ contribution

S.M. designed the study, made the first draft, updated the manuscript, and reviewed the final draft.

## Conflicts of interest disclosure

The authors declare that they have no financial conflict of interest with regard to the content of this report.

## Research registration unique identifying number (UIN)

Not applicable.

## Guarantor

Sumira Malik.
